# Application of Microsatellites in Genetic Diversity Analysis and Population Discrimination of *Coilia nasus* from the Yangtze River

**DOI:** 10.3390/ani16030459

**Published:** 2026-02-01

**Authors:** Yu Zhang, Wenrong Feng, Jia Wei, Jie Liu, Jizhou Lv, Yongkai Tang

**Affiliations:** 1 Wuxi Fisheries College, Nanjing Agricultural University, Wuxi 214081, China; 15005128042@163.com (Y.Z.); 13153030538@163.com (J.W.); 2 Key Laboratory of Freshwater Fisheries and Germplasm Resources Utilization, Ministry of Agriculture and Rural Affairs, Freshwater Fisheries Research Center, Chinese Academy of Fishery Sciences, Wuxi 214081, China; fengwenrong@ffrc.cn (W.F.); maydayjiel@163.com (J.L.); 3 Chinese Academy of Quality and Inspection & Testing, Beijing 100176, China; ljzffff@163.com

**Keywords:** *Coilia nasus*, genetic diversity, microsatellite markers, discriminant function

## Abstract

The genetic diversity and population structure of five tapertail anchovy (*Coilia nasus*) populations—four wild populations from the Yangtze River (Taizhou, Anqing, Shanghai, Hukou) and one cultured population from Yangzhong—were analyzed using 18 highly polymorphic microsatellite loci. All loci exhibited high polymorphism, with genetic parameters as follows: mean number of alleles = 20.567, expected heterozygosity = 13.506, Shannon information index = 2.743, and polymorphic information content = 0.9624. F-statistics ranged from 0.02898 to 0.05714, indicating varying degrees of genetic differentiation between all populations. Analysis of molecular variance revealed that 4% of the total genetic variation was attributable to differences among populations, 23% to variation among individuals within populations, and 73% to within-individual genetic variation. A UPGMA phylogenetic tree based on Nei’s genetic distance showed that the Shanghai population clustered first with Anqing, followed by Taizhou, Hukou, and finally Yangzhong. Additionally, discriminant functions developed from microsatellite data enabled accurate population assignment for all individuals. These findings provide critical insights into the genetic relationships and structure of *C. nasus* populations, offering valuable implications for their conservation and management.

## Simple Summary

Understanding the genetic connections and population characteristics of tapertail anchovy (*Coilia nasus*) is crucial for protecting this species and its living resources. This study examined five *Coilia nasus* populations—four wild populations living in the Yangtze River and one cultured population—by analyzing special genetic markers with high variability. The results showed high genetic diversity among these groups, with different degrees of genetic differences between them. Most genetic variation existed within individual fish, while variation between groups was small. Genetic analysis also revealed the evolutionary relationships between the groups: the Shanghai population clustered first with Anqing, followed by Taizhou, Hukou, and finally Yangzhong. We also developed a method to accurately identify which population each fish belongs to. These findings clarify the genetic links of *Coilia nasus* populations and provide important guidance for protecting and properly managing this species’ resources.

## 1. Introduction

Tapertail anchovy (*Coilia nasus*), commonly known as knife fish, phoenix tail fish, or hair flower fish, belongs to the order Clupeiformes, family Engraulidae, and genus *Coilia*. This species is predominantly found in the northwestern and western Pacific Ocean. In China, *C. nasus* is widely distributed across the Yellow Sea, Bohai Sea, East China Sea, and in various connected rivers and lakes, such as the Yangtze River, Yellow River, Liao River, and Qiantang River. It is considered a common small-to-medium-sized commercial fish in China [1]. Historically, *C. nasus* was abundant in terms of resources and catch, with its flesh—known for its tender texture and high fat content—being highly prized. It was even regarded as one of the “Three Delicacies of the Yangtze River”. Threatened by overexploitation and habitat loss in the early 21st century, the population of *C. nasus* has declined significantly [2]. As a result, *C. nasus* has been listed on the IUCN Red List of Endangered Species [3].

In recent years, with the implementation of national policies such as the “Yangtze River Protection Law” and the “10-Year Fishing Ban on the Yangtze River” catches of wild *C. nasus* have been prohibited. As a result, the demand for *C. nasus* is mainly met through aquaculture. Due to the high price of wild-caught *C. nasus*, illegal fishing still occurs. The emergence of traceability technologies has helped regulate and ensure the authenticity of these products. However, current assessments of the genetic structure and diversity of wild and farmed populations are limited, and research on seafood traceability is still in its infancy in China [4]. As a result, pinpointing and tracing the origin of *C. nasus* from the Yangtze River has become a critical issue that must be addressed.

Research on the genetic diversity of *C. nasus* has only been initiated in recent decades. Cheng et al. [5] analyzed the genetic polymorphism and genetic relationships of *C. nasus* species using mitochondrial cytochrome b gene fragments as molecular markers. Additionally, Yang et al. [6] used the full sequence of the mitochondrial control region as molecular markers to analyze the genetic structure of *C. nasus* populations in the Yangtze River estuary and adjacent areas. However, there is a notable degree of genetic differentiation among different populations of *C. nasus*. These findings suggest that the resources of *C. nasus* have been compromised, and its genetic pool has diminished, highlighting the urgent need for enhanced conservation efforts and further research. The genetic structure and diversity of a population serve as the theoretical foundation and crucial reference for the rational development and sustainable utilization of its resources. *C. nasus* is widely distributed and has formed numerous geographic populations. Due to environmental changes and adaptation to different habitats, these geographic populations exhibit variations. However, the extent of differences between these populations remains poorly understood, and research on traceability technologies is scarce.

Microsatellite DNA is one of the most important molecular markers currently used to construct genetic maps. There are linked relationships between genes, and some genes can form linkage groups that can be independently separated from other linkage groups. Researchers can obtain the order and relative distance of individual genes on the chromosomes based on the genetic distances between them, and localize multiple genes directly on the chromosomes, thus constituting a physical map. Zhang et al. [7] constructed a high-density linkage map using 164 female *Epinephelus fuscoguttatus* and their female parents Currently, microsatellite markers have been successfully applied in parentage identification across various species [8]. This method, particularly in forensic science, is considered one of the most accurate techniques for individual identification and parentage testing. It has also been widely used in marine species. Zhang et al. [9] studied the relationship between the accuracy of microsatellite-based parentage identification, the identification ability, and the size of the candidate parent population in Hucho taimen, revealing that the identification ability decreased as the size of the candidate parent population increased. Yang et al. [10] used 11 pairs of developed microsatellite primers to tag parents and established a systematic evaluation method of “tagging parents—release and re-capture—paternity identification”, with high identification accuracy.

Therefore, in this study, a systematic analysis of *C. nasus* population was carried out through microsatellite markers, and the discriminant function was constructed using microsatellite data. The objective was to analyze the population construction of *C. nasus* from the genetic diversity level, population genetic structure and population discrimination perspectives. This will help to promote the conservation of *C. nasus* germplasm resources and accurately locate the origin of *C. nasus* at the molecular level, which will help people to protect wild *C. nasus*.

## 2. Materials and Methods

### 2.1. Materials

**Figure 1:**
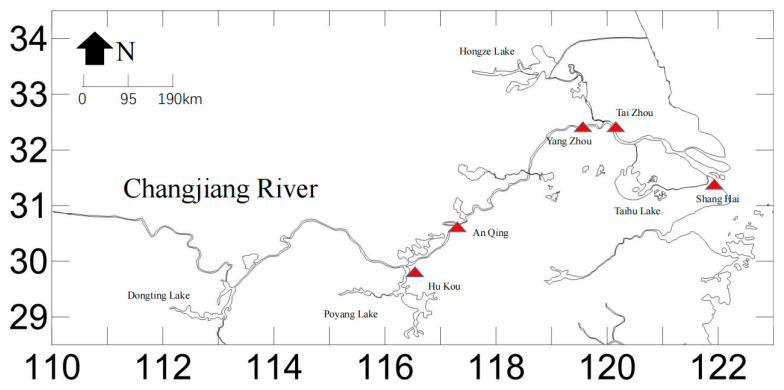
Locations of five sampling *C. nasus* stocks. Note: the red triangle represents the sample collection location.

From April to June 2023, wild *C. nasus* were sequentially collected from the Taizhou (TZ), Anqing (AQ), Shanghai (SH), and Hukou (HK) sections of the Yangtze River, as well as cultured *C. nasus* at the Yangzhong Scientific Research and Experimental Base of the Freshwater Fisheries Research Center, Chinese Academy of Fishery Sciences (YZ). The sampling locations are presented in Figure 1. 32 healthy individuals were randomly selected from each group, and a portion of their caudal fins was preserved in 95% ethanol solution. After assigning identification numbers, the samples were stored at −20 °C for future experimental studies.

### 2.2. Methods

Genomic DNA extraction was performed according to the OMEGA kit instructions. The quality and concentration of the extracted DNA were assessed using agarose gel electrophoresis and a UV-Vis spectrophotometer. Electrophoresis on 1.5% agarose gel indicated no degradation or tailing, with clear bands, and the samples were stored at −20 °C for future study.

Based on the microsatellite sequences and primer information of *C. nasus* in the Yangtze River reported in the literature, 24 pairs of microsatellite primers with high polymorphism were finally selected [11,12]. Microsatellite primers were modified by fluorescent labeling at the 5′ end: forward primers were labeled with FAM (blue fluorescence) or with HEX (green fluorescence). All reverse primers remain unlabeled.

The PCR reaction mixture was prepared with a total volume of 20 μL, consisting of 10 μL 2× Rapid Taq Master Mix, 1 μL of Primer F, 1 μL of Primer R, 1 μL of cDNA, and 7 μL of ddH_2_O. The PCR amplification program was as follows: 3 min of pre-denaturation at 95 °C, followed by 35 cycles of denaturation at 95 °C for 15 s, annealing at 60 °C for 15 s, and extension at 72 °C for 15 s, with a final extension at 72 °C for 5 min and termination at 4 °C. The final primer concentration in the PCR reaction is 5 μM. The amplified PCR products were detected by capillary electrophoresis on the ABI-3730XL gene analyzer (ABI Biological Thermo Fisher Scientific, California, CA, USA). Each CE sample contained 1 μL of PCR product, 0.4 μL of Genescan-500 molecular quality standard and 20 μL of deionized formamide (ABI Biological Company, USA). The microsatellite loci are amplified separately for each sample. In the process of capillary electrophoresis, the fluorescence signals of PCR products and Genescan-500 molecular quality standards were automatically stored by gene analyzer. Each sample is individually loaded into a capillary electrophoresis analyzer during operation. After screening, 18 pairs of primers with good amplification effect and high polymorphism were obtained. Microsatellite primer information is provided in Table 1. Primer synthesis and SSR genotyping were completed by Shanghai Yixin Biotechnology Co., Ltd. (Shanghai, China).

### 2.3. Data Analysis

The GenAlEx 6.5 software was used to calculate and analyze Observed number of alleles (*Na*), Observed heterozygosity (*Ho*), Expected heterozygosity (*He*), and HWE index for polymorphic microsatellite loci. The POPGENE (version 32) software was used to calculate allele frequencies for each locus, *N_m_* between populations, Within-population inbreeding coefficient (*Fis*), followed by UPGMA cluster analysis. The matrix of pairwise *F_ST_* values was calculated using Arlequin (version 3.5.2.2) software. AMOVA analysis is also conducted using GenAlEx 6.5 software. The PowerMarker V3.25 software was employed to conduct HWE tests for each locus. The polymorphic information content (PIC) for each microsatellite locus was calculated. Genetic distance between populations was estimated, and Structure 2.3.4 was utilized to perform clustering of different wild populations. Additionally, clustering analysis of wild and farmed populations was carried out, using a mixed model to uncover hidden population genetic structures.

A stepwise linear discriminant analysis (LDA) of 36 genotypic fragment length data sets amplified by 18 pairs of SSR primers from five *C. nasus* populations (TZ, SH, AQ, HK and YZ) was performed using SPSS 27 software. In the experiment, 160 *C. nasus* (32 from each population) were selected, 100 individuals (20 from each population) were randomly selected to construct the discriminant model by stepwise discriminant method, and the remaining 60 individuals (12 from each population) were substituted into the model for back generation validation in order to evaluate the accuracy of the discriminant model.

## 3. Results

### 3.1. Microsatellite Locus Polymorphism and Population Genetic Diversity

As shown in Table 2, a total of 370 alleles were detected across 18 loci, with the number of alleles ranging from 15 to 28 per locus. On average, each locus had 20 alleles. The effective number of alleles (*Ne*) ranged from 10.36 to 19.414, with an average of 13.506. I ranged from 2.498 to 3.123, with an average of 2.742. *Ho* ranged from 0.488 to 0.969, with an average of 0.71. The PIC ranged from 0.9412 to 0.9791, with an average of 0.9624. According to the standard set by Botstein et al. [13], a PIC value ≥ 0.5 indicates high polymorphism. All 18 loci in this study were highly polymorphic, suggesting that these loci provide rich genetic information and can be used for assessing the genetic diversity of *C. nasus* populations. F-test results revealed that among the 18 loci, one locus had a negative inbreeding coefficient, while the remaining 17 loci exhibited positive values, indicating a relatively high level of inbreeding. The gene flow at each locus was greater than 1, with an average gene flow of 5.625.

As shown in Table 3, the SH population exhibited the lowest values for *Na* (19.111) and *Ho* (0.642), while the HK population had the lowest values for *N_e_* (11.903), I (2.626), and *He* (0.903). In contrast, the YZ population had the highest values for *Na* (22.5), *N_e_* (15.712), I (2.877), *Ho* (0.788), and *He* (0.931). The PIC values for all populations were greater than 0.5, indicating a high level of genetic polymorphism.

### 3.2. Population Genetic Differentiation and Genetic Distance

As shown in Table 4, according to Wright’s recommendations, the genetic differentiation level between the AQ and SH populations was the lowest (*F_ST_* = 0.02898, *p* < 0.01), while the genetic differentiation level between the AQ and HK populations was the highest (*F_ST_* = 0.05714, *p* < 0.01).

As shown in Table 5, variation between populations accounted for 4% of the total variation, which was statistically significant (*p* < 0.01). Variation among individuals within populations accounted for 23% of the total variation (*p* < 0.01), while genetic variation within individuals accounted for 73% of the total variation (*p* < 0.01). This indicates that genetic variation between individuals is much greater than that between populations, and the majority of genetic variation exists within individuals.

**Figure 2:**
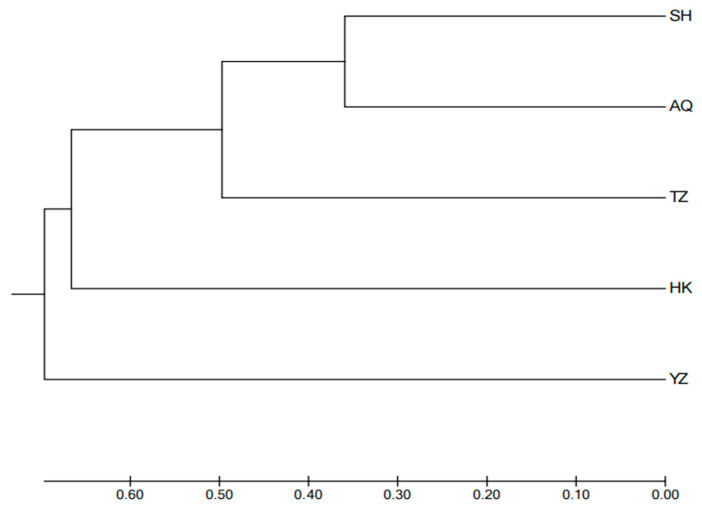
A UPGMA clustering tree for five populations of *C. nasus* based on Nei’s genetic distance. Note: SH, Shanghai; AQ, Anqing; TZ, Taizhou; HK, Hukou; YZ, cultivated population; the scale bar in the image represents the unit length of Nei genetic distance.

As shown in Table 6, the Nei’s genetic distances between the five populations ranged from 0.719 to 1.898. The largest Nei’s genetic distance was observed between the TZ and YZ populations (1.898), indicating the lowest genetic similarity (0.150). The smallest Nei’s genetic distance was observed between the SH and AQ populations (0.719), indicating the highest genetic similarity (0.487). The UPGMA phylogenetic tree, constructed based on Nei’s genetic distance (Figure 2), shows that the SH population first clustered with the AQ population, then with the TZ population, followed by the HK population, and finally with the YZ population.

### 3.3. Analysis of Population Genetic Structure

**Figure 3:**
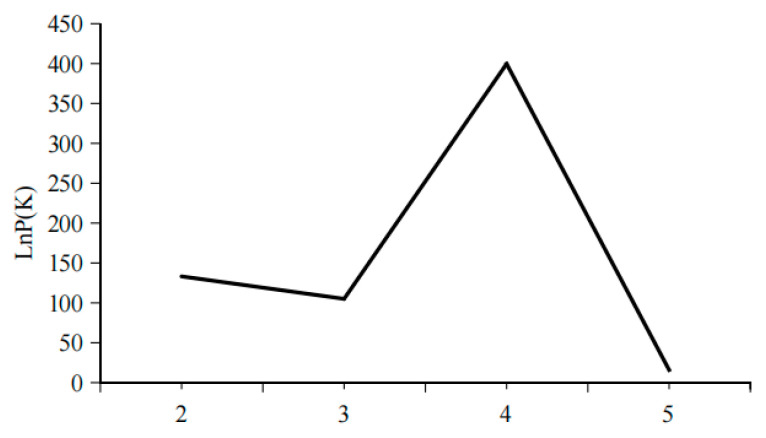
Trend chart of inter group K values and their corresponding parameters.

**Figure 4:**
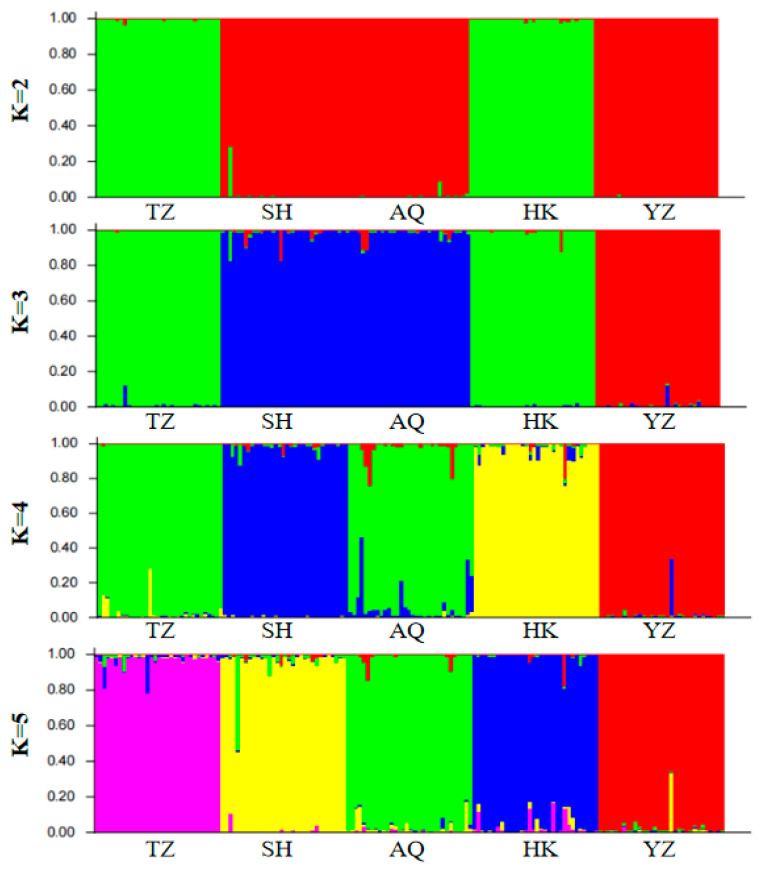
Structure genetic cluster analysis for *C. nasus*. Note: TZ, Taizhou; SH, Shanghai; AQ, Anqing; HK, Hukou; YZ, cultivated population.

The optimal number of populations (K) was determined using Structure Harvester’s Evanno method (Figure 3). Based on the analysis of 18 microsatellite loci, the Structure bar plot for the five *C. nasus* populations (Figure 4) was obtained. The most optimal population division occurred when K = 3. The individual assignment patterns in the Structure bar plot indicated that the *C. nasus* populations could be divided into three freely mating groups. The TZ, SH, and AQ populations were primarily assigned to the same group, while the HK and YZ populations were distinctly separated from the other three populations.

### 3.4. Discriminant Analysis of Principal Components

**Figure 5:**
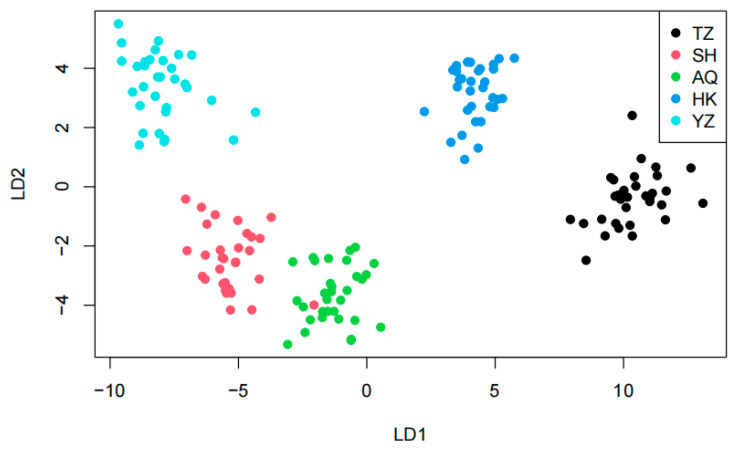
Genetic structure of five *C. nasus* populations based on Discriminant Analysis of Principal Components. Note: TZ, Taizhou; SH, Shanghai; AQ, Anqing; HK, Hukou; YZ, cultivated population.

Perform DAPC analysis using the adegenet package in R 4.3.1. Obtained a scatter plot of the population distribution of the *C. nasus*, which is used to understand the genetic structure of the population and verify the rationality of population division (Figure 5).

### 3.5. Discriminate Analysis

LDA was conducted on the data set of 36 SSR genotypic fragment lengths from *C. nasus* using SPSS 27 software. A discriminant function was obtained with 11 morphometric indicators as independent variables, which included 11 SSR genotypic fragment lengths components (Table 7). The result showed that the first data set of primer Cnas05 exhibited the highest coefficients among the 11 morphometric indicators, followed by the first set of data for Cnas09 and Cnas15. in second and third place respectively.

**Figure 6:**
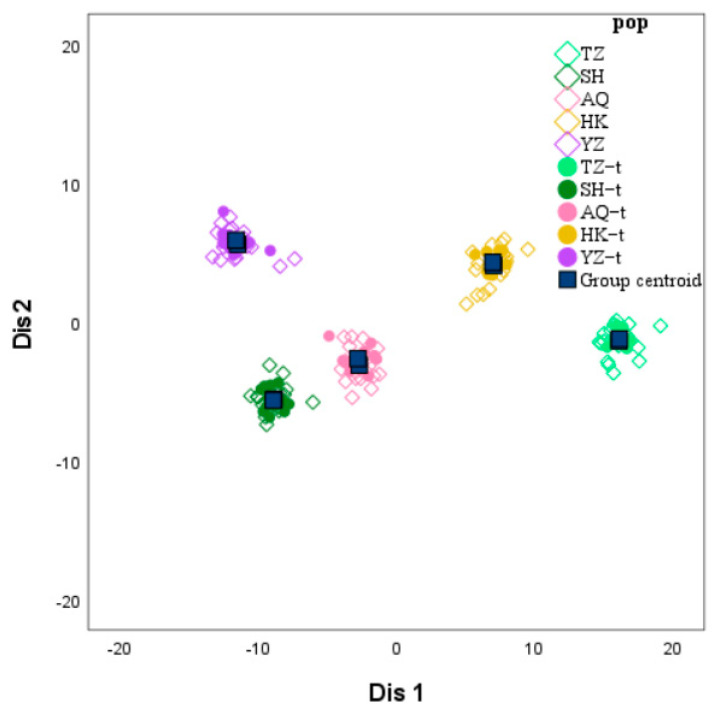
Scatterplot of scores based on the first two canonical discriminant functions for ten groups of TZ, SH, AQ, HK, YZ, TZ−t, SH−t, AQ−t, HK−t and YZ−t. Note: TZ, Taizhou; SH, Shanghai; AQ, Anqing; HK, Hukou; YZ, cultivated population; TZ−t, Taizhou−test; SH−t, Shanghai−test; AQ−t, Anqing−test; HK−t, Hukou−test; YZ−t, cultivated population−test.

The remaining 60 of the five *C. nasus* populations (named TZ−t, Taizhou−test; SH−t, Shanghai−test; AQ−t, Anqing−test; HK−t, Hukou−test; YZ−t, cultivated population-test) are brought back to the above formula and scatter plot of discrimination are shown in Figure 6. The results showed that the overall discrimination success rate was 100% among the remaining 60 of the five *C. nasus* populations (Table 8). It can be seen that the discrimination data based on the length composition of the SSR genotypic fragments in the caudal fins of *C. nasus* can be used to distinguish *C. nasus* from different sections of the Yangtze River.

## 4. Discussion

Microsatellite markers have become a popular tool for detecting genetic variation in aquatic species due to their co-occurrence, ease of detection, high polymorphism, stability, and minimal DNA quality requirements. In the present study, 18 microsatellite loci were chosen to investigate the genetic diversity and structure of four wild populations and one breeding population of *C. nasus*. The analysis revealed average values of 20.567 for *Na*, 13.506 for *Ne*, and high polymorphism with PIC averaging 0.919, indicating strong genetic diversity. All 21 microsatellite loci had PIC values exceeding 0.5, confirming their high polymorphism. These markers are suitable for assessing genetic diversity in *C. nasus* populations and can be applied in selection and breeding programs. The results from this study will provide valuable data for monitoring genetic variation in *C. nasus* populations.

*Na*, *Ne*, I, *Ho*, *He*, and PIC are key parameters used to assess the genetic diversity of a population, and they are all positively correlated with it. It is generally believed that the closer *Na* is to the absolute value of *Ne* indicates that the population alleles are more evenly distributed and have higher genetic diversity [14]. In the current study, *Na* was found to be greater than *Ne* in all five populations of *C. nasus*, indicating that the alleles were unevenly distributed across these populations. This pattern has also been observed in species like cipangopaludina chinensis [14] and macrobrachium rosenbergii [15]. The index I reflects both the genetic richness and evenness of a population [16]. For instance, the average I-value of nine populations of tachypleus tridentatus was 1.39, indicating high genetic diversity in these populations [17]. In the case of cipangopaludina chinensis, I-values ranged from 0.412 to 1.226, also showing significant genetic diversity [14]. In this study, the I-values of the five *C. nasus* populations ranged from 2.626 to 2.794, with an average of 2.743, reflecting high genetic diversity in all five populations. While *Ho* can provide insight into genetic diversity, *He* is less influenced by sample size and is therefore often considered a more reliable measure of population diversity [18]. A higher *He* value generally indicates greater genetic diversity. For example, *He* values for 14 populations of pelteobagrus fulvidraco ranged from 0.358 to 0.749, suggesting high genetic diversity [19]. In this study, the *He* values for the five populations of *C. nasus* ranged from 0.903 to 0.931, indicating high genetic diversity across all populations. Furthermore, when *He* exceeds *Ho*, as seen in nine populations in a prior study, it suggests the potential presence of heterozygous deletions, possibly due to rare genotypes, dummy alleles, artificial selection, or inbreeding [20]. Similar patterns were observed in the study of six cyprinus carpio populations by Dong et al. [21]. The inbreeding coefficient within population (*Fis*) is a core parameter in population genetics for quantifying the level of inbreeding among individuals within a population. In the present study, the *Fis* values of the *C. nasus* populations ranged from 0.1 to 0.3, indicating a mild level of inbreeding with a slight deficit of heterozygotes, which exerted a negligible impact on the genetic composition of the populations. This pattern could be attributed to the anadromous migratory behavior of *C. nasus*: annual migrations facilitate genetic exchange among different geographic populations, thereby mitigating the degree of inbreeding within individual populations.

A *F_ST_* value of less than 0.05 indicates a low level of genetic differentiation between populations, while values between 0.05 and 0.15 suggest moderate differentiation, and values between 0.15 and 0.25 indicate a high level of differentiation [19]. In the present study, the *F_ST_* values ranged from 0.2898 to 0.5714, indicating a low level of genetic differentiation among most populations. Notably, the *F_ST_* values between the HK population with the SH, AQ and YZ populations were all greater than 0.05, which demonstrated a moderate degree of genetic differentiation between the Hukou population and the aforementioned populations (*p* < 0.01). This phenomenon could be attributed to the fact that the HK population inhabits Poyang Lake permanently, leading to limited genetic exchange with other populations. In contrast, the remaining populations undertake migratory reproduction along the Yangtze River, thus facilitating relatively frequent genetic communication. Gene flow plays a key role in maintaining low genetic differentiation between populations. Generally, if *N_m_* is less than 1, it suggests potential genetic segregation, while *N_m_* more than 1 means genetic differentiation is not significant, and *N_m_* more than 4 indicates very minimal differentiation [22]. In this study, all populations had *N_m_* values more than 4 (ranging from 6.449 to 10.858), which indicates frequent gene flow between populations and low genetic differentiation. The *C. nasus* is a migratory fish species, and their migration route is from the Yellow Sea to the mouth of the Yangtze River in Shanghai, and then upstream along the main channel of the Yangtze River. We collected samples along this route. Most *C. nasus* are migratory, but there are also some that stay in large lakes along the way, and the annual migration season promotes their genetic exchange [23]. The AMOVA analysis revealed that 4% of the genetic variation came from differences within populations, 23% from differences between individuals, and 73% from differences within individuals. This also supports the conclusion that genetic differentiation among the five populations of *C. nasus* is low. The standardized genetic distances, calculated using Nei’s method, reflect the degree of genetic differentiation, while the genetic similarity coefficients show the relatedness between populations, with the two being inversely proportional [24]. In this study, genetic distances between populations ranged from 0.719 to 1.898, with similarity coefficients varying between 0.150 and 0.487. The SH and AQ populations were the most genetically similar, with the smallest genetic distance (0.719) and highest similarity coefficient (0.487). In contrast, the YZ population had larger genetic distances from other populations (1.056 to 1.898) and lower similarity coefficients (0.150 to 0.348), reflecting its distinctness from the other populations. This could also be due to other groups being wild populations. According to the UPGMA clustering tree based on Nei’s genetic distance, the SH group clustered first with the AQ group, then with the TZ group, followed by the HK group, and finally the YZ group. Similarly, the PCoA results showed that the SH and AQ groups clustered together, while the YZ group was distinct.

Structure is considered an ideal method for analyzing population genetic structure, as its computational process does not require prior knowledge of the genetic background of the populations. In this study, K values ranging from 2 to 5 were selected, with the number of repetitions set to twenty. The trends of K values and the mean of the related statistic Var[LnP(K)] were analyzed (Figure 3). A clear inflection point was detected at K = 3, indicating that the optimal estimated number of theoretical populations is three. Examination of the structure plots for different K values (primarily at K = 3) revealed that the genetic structures of SH and AQ are relatively close, while those of TZ and HK are also similar. In contrast, the genetic structure of YZ is distinctly distant from the other four populations, with only a small number of individuals exhibit cross mixing. From a geographical perspective, SH and AQ are both located along the main channel of the Yangtze River, while TZ and HK are situated at the confluence of Poyang Lake and Hongze Lake with the Yangtze River, respectively. YZ represents an artificially cultured population. Their geographical distribution is consistent with the genetic structure inferred by Structure analysis. DAPC does not rely on assumptions of Hardy–Weinberg equilibrium or linkage equilibrium and can provide a robust, model-free assessment of population structure. As shown in Figure 5, individuals from SH and AQ exhibit some degree of overlap, whereas populations TZ and HK are closely clustered. The YZ population is clearly separated from the other four populations, with no observed individual-level admixture. The inclusion of DAPC strengthens the inference of genetic clustering and validates that the division of *C. nasus* populations into three genetic groups is biologically meaningful.

To investigate the population structure of *C. nasus*, Xu et al. [25] employed landmark-based geometric morphometric analysis to quantify otolith morphological characteristics and demonstrated efficacy in distinguishing *C. nasus* across distinct aquatic habitats from the Yangtze River. Otolith microchemistry also can be a good way to clearly determined the habitats of *C. nasus* [26]. In addition, muscle mineral elements can be used to determine fish habitat [27]. In the current study, we adopted a molecular-level approach utilizing microsatellite markers to differentiate *C. nasus* populations from various geographical locations. Studies have shown that SNP markers can be used to genetically discriminate fish populations. RA Del [28] used SNP markers for population genetic analysis of farmed mussels, and more than 90% of the individuals were successfully localized to their place of origin; Zhang et al. [29] used microsatellite DNA analysis to determine the origin of 190 farmed escaped Atlantic salmon. In our study, the results demonstrated 100% classification accuracy, indicating that this molecular approach effectively identifies the geographical origins of *C. nasus* and provides a reliable method for provenance determination. This method enables market regulatory authorities to effectively trace the geographic origin of wild *C. nasus*, thereby helping to reduce illegal fishing activities.

## 5. Conclusions

In summary, the genetic diversity and genetic structure of four wild populations and one cultured population of *C. nasus* were evaluated using 18 highly polymorphic microsatellite loci in the present study. PIC > 0.5 indicated that the 18 microsatellite loci were highly genetically polymorphic. The results of I and *He* showed that *C. nasus* have high genetic diversity. *F_ST_*, *N_m_* and AMOVA indicated low levels of genetic differentiation among the five populations. The constructed UPGMA reflected the genetic distance relationship among the five populations, with the distances in the order of SH, AQ, TZ, HK and YZ. Discriminant functions were constructed via LDA method and was able to distinguish 100% between the five populations of *C. nasus*. LDA successfully constructed discriminant functions that achieved 100% separation among the five *C. nasus* populations. This study lays the foundation for accurately locating and tracing the origin of *C. nasus* and the population classification of *C. nasus* in the Yangtze River.

## Figures and Tables

**Table 1:** 18 pairs of microsatellite primers of *C. nasus*.

Locus	Primer Sequence (5′—3′)	Fragment Size (bp)	Fluorescence
DJ41	GTGATGTTGTTGAACCTGAG	126–186	5′FAM
	CCGTCCTTTACTCTTTGTCT	140–198	5′FAM
DJ578	TGTCACACCGAGTAATGTC	127–161	5′HEX
	AGAGGATGAGAGATAGAAGGA	181–209	5′FAM
Cnas01	TGCTATCTGTGATGATGCTACG	275–299	5′HEX
	GAATACCTGCCCTTGTTTCTTG	173–207	5′FAM
Cnas02	ATGGCAGGGAGGACAGGTAT	191–201	5′FAM
	TGTGGCAAGCAGTGGTAAGT	189–259	5′HEX
Cnas03	AACAGTATGGTCTATGTGGGAA	247–291	5′HEX
	ACTTGTGGAATTGTGTGGTATT	262–306	5′FAM
Cnas04	GTATGTTTGTGAGGGTATGGGA	136–160	5′HEX
	GTGAGCAAGGAACGGATAGTAA	155–181	5′HEX
Cnas05	TACACAGACACGAATGGCACAA	282–316	5′HEX
	TCCACAAACTCGTAGGCAATCC	198–211	5′FAM
Cnas06	TTCCTCTACTGGTCACAATGCT	169–218	5′FAM
	ATGTCTCCACCTCCAACAGATT	152–205	5′FAM
Cnas07	CATTCTTTCCTCCCATCCCATA	168–222	5′HEX
	GACTCCTTCCAAGACCCACTGA	205–260	5′FAM
Cnas08	TTACATTAGGGGCTTCTGAGGC	126–186	5′FAM
	AGACCCAGGACACTTCCCAACC	140–198	5′FAM
Cnas09	ATGATTGGTGTTCCTTTATGCT	127–161	5′HEX
	TCCGAGAGTTCTTCCAGGTGAG	181–209	5′FAM
Cnas11	AGGTGGTAAAAGACCCAAAGAA	275–299	5′HEX
	TGTTTTCCCTCAATCCATCATA	173–207	5′FAM
Cnas15	TGGTAATGGCACTAATGGTATG	191–201	5′FAM
	TAAATCAACCAACCAAGAAACA	189–259	5′HEX
Ce02	CAGGCAATGTATTATTCAAAGG	247–291	5′HEX
	TGACTGGAATCAGGCAGCACTT	262–306	5′FAM
Ce03	CTTCATACAGTATTAGGACCAGA	136–160	5′HEX
	ACTTCAGTCTGCTTTGGAATGTG	155–181	5′HEX
Ce04	CAATGAGGTACGTTATTCACAGAA	282–316	5′HEX
	TGCAGACCAGATCTACTGAGGCTCC	198–211	5′FAM
Ce06	GTACACTCACTTAACCTGTGCTTCA	169–218	5′FAM
	ATTTTCCCCTCCATTAGAAGACATC	152–205	5′FAM
Ce07	CGTAGTATTCAATGGCGTTGTTC	168–222	5′HEX
	GTACTGCTACACCTCCCTTTCAC	205–260	5′FAM

**Table 2:** Genetic diversity parameters and the estimates of gene flow among 18 loci of *C. nasus*.

Locus	*Na*	*N_e_*	I	*Ho*	*He*	PIC	*Fis*	*F_ST_*	*N_m_*
DJ41	16.2	10.722	2.525	0.969	0.905	0.948	−0.070	0.048	4.997
DJ578	21.8	12.602	2.739	0.594	0.909	0.9569	0.347	0.052	4.565
Cnas01	20.2	13.371	2.741	0.738	0.921	0.9412	0.199	0.024	10.104
Cnas02	17	11.054	2.578	0.744	0.907	0.967	0.180	0.063	3.715
Cnas03	21	14.035	2.811	0.806	0.926	0.9769	0.130	0.052	4.529
Cnas04	28.4	18.994	3.123	0.85	0.946	0.9678	0.101	0.024	10.206
Cnas05	17.4	10.36	2.547	0.688	0.902	0.9665	0.238	0.068	3.441
Cnas06	22.4	15.434	2.887	0.588	0.933	0.9791	0.370	0.048	4.974
Cnas07	26	16.474	2.978	0.644	0.934	0.9653	0.311	0.034	7.179
Cnas08	26.8	19.414	3.109	0.838	0.948	0.9783	0.116	0.032	7.679
Cnas09	16.4	10.77	2.532	0.619	0.905	0.953	0.317	0.052	4.564
Cnas11	23.8	15.388	2.875	0.763	0.926	0.9673	0.176	0.044	5.461
Cnas15	15.4	11.579	2.523	0.594	0.906	0.9427	0.345	0.042	5.749
Ce02	25.8	18.273	3.049	0.663	0.944	0.9791	0.298	0.036	6.677
Ce03	17.4	10.221	2.498	0.581	0.89	0.9597	0.347	0.074	3.122
Ce04	17.4	11.321	2.589	0.844	0.91	0.9479	0.072	0.043	5.597
Ce06	17.8	12.008	2.643	0.769	0.916	0.9651	0.161	0.052	4.555
Ce07	19	11.082	2.622	0.488	0.909	0.9622	0.463	0.057	4.139
Mean	20.567	13.506	2.743	0.71	0.919	0.9624	0.228	0.047	5.625

Note: *Na*, Observed number of alleles; *Ne*, Effective number of alleles; I, Shannon’s Information index; *Ho*, Observed heterozygosity; *He*, Expected heterozygosity; PIC, Polymorphic information content; *Fis*, Within-population inbreeding coefficient; *F_ST_*, Fixation index; *N_m_*, Gene flow [*N_m_* = 0.25(1 − *F_ST_*)/*F_ST_*].

**Table 3:** Genetic diversity of five populations of *C. nasus*.

Parameter	TZ	SH	AQ	HK	YZ	Mean
*N*	32	32	32	32	32	32
*Na*	21.444	19.111	20.167	19.611	22.5	20.5666
*N_e_*	13.802	12.909	13.203	11.903	15.712	13.5058
I	2.794	2.692	2.726	2.626	2.877	2.743
*Ho*	0.674	0.642	0.707	0.738	0.788	0.7098
*He*	0.925	0.917	0.918	0.903	0.931	0.9188
*Fis*	0.271	0.299	0.230	0.183	0.153	0.2272

Note: TZ, Taizhou; SH, Shanghai; AQ, Anqing; HK, Hukou; YZ, cultivated population; *N*, number; *Na*, Observed number of alleles; *Ne*, Effective number of alleles; I, Shannon’s information index; *Ho*, Observed heterozygosity; *He*, Expected heterozygosity; *Fis*, Within-population inbreeding coefficient.

**Table 4:** Matrix of pair-wise *F_ST_* values between five populations of *C. nasus*.

*F_ST_*	TZ	SH	AQ	HK	YZ
TZ	0.00000				
SH	0.03903 *	0.00000			
AQ	0.03326 *	0.02898 *	0.00000		
HK	0.04691 *	0.05133 *	0.05714 *	0.00000	
YZ	0.04734 *	0.03862 *	0.03567 *	0.05051 *	0.00000

Note: TZ, Taizhou; SH, Shanghai; AQ, Anqing; HK, Hukou; YZ, cultivated population; *: very significance (*p* < 0.01).

**Table 5:** AMOVA analysis among five populations of *C. nasus*.

Source of Variation	df	Sum of Square	Variance Components	Percentage of Variation (%)
Among populations	4	130.031	0.344	4 *
Among individuals within populations	155	1623.609	2.044	23 *
Within individuals	160	1022.000	6.388	73 *
Total variation	319	2775.641	8.775	

Note: * very significance (*p* < 0.01).

**Table 6:** Nei’s genetic similarity (above diagonal) and genetic distance (below diagonal) of five populations of *C. nasus*.

	TZ	SH	AQ	HK	YZ
TZ	-	0.338	0.405	0.304	0.150
SH	1.085	-	0.487	0.286	0.316
AQ	0.903	0.719	-	0.212	0.348
HK	1.192	1.253	1.551	-	0.232
YZ	1.898	1.153	1.056	1.462	-

Note: TZ, Taizhou; SH, Shanghai; AQ, Anqing; HK, Hukou; YZ, cultivated population.

**Table 7:** Fisher’s linear discriminant function for fragment lengths of 36 SSR genotypes from five populations of *C. nasus*.

Morphometric Indicator	TZ	SH	AQ	HK	YZ
X_1_	1.849	2.254	2.131	2.414	2.365
X_2_	0.571	0.802	0.671	0.604	0.818
X_3_	1.042	2.043	1.616	1.597	2.192
X_4_	4.638	5.950	5.263	5.538	6.512
X_5_	1.082	0.391	0.861	0.953	0.869
X_6_	0.636	0.371	0.487	0.577	0.508
X_7_	1.309	0.957	0.942	1.135	0.898
X_8_	4.241	3.388	3.315	3.644	3.034
X_9_	3.299	3.270	3.258	3.294	3.687
X_10_	0.028	−0.267	−0.201	−0.052	−0.422
X_10_	0.028	−0.267	−0.201	−0.052	−0.422
X_11_	3.229	2.254	2.219	2.966	1.951
digital	−3074.735	−2941.321	−2678.988	−3230.871	−3288.946

Note: X_1_:, second set of data for DJ41; X_2_: second set of data for DJ578; X_3_: second set of data for Cnas03; X_4_: first set of data for Cnas05; X_5_: first set of data for Cnas06; X_6_: second set of data for Cnas07; X_7_: first set of data for Cnas08; X_8_: first set of data for Cnas09; X_9_: first set of data for Cnas15; X_10_: first set of data for Ce02; X_11_: first set of data for Ce06.

**Table 8:** Results of microsatellite discriminant analysis of five populations of *C. nasus*.

	TZ	SH	AQ	HK	YZ	Correct Rate
TZ−t	12	0	0	0	0	100%
SH−t	0	12	0	0	0	100%
AQ−t	0	0	12	0	0	100%
HK−t	0	0	0	12	0	100%
YZ−t	0	0	0	0	12	100%

Note: TZ, Taizhou; SH, Shanghai; AQ, Anqing; HK, Hukou; YZ, cultivated population; TZ−t, Taizhou−test; SH−t, Shanghai−test; AQ−t, Anqing−test; HK−t, Hukou−test; YZ−t, cultivated population−test.

## Data Availability

Data will be made available on request.

## Abbreviations

The following abbreviations are used in this manuscript:

TZ	Taizhou
AQ	Anqing
SH	Shanghai
HK	Hukou
YZ	Yangzhong
*Na*	Observed number of alleles
*N_e_*	Effective number of alleles
I	Shannon’s Information index
*Ho*	Observed heterozygosity
*He*	Expected heterozygosity
PIC	Polymorphic information content
*Fis*	Within-population inbreeding coefficient
*F_ST_*	Fixation index
*N_m_*	Gene flow
K	The optimal number of populations
TZ−t	Taizhou−test
AQ−t	Anqing−test
SH−t	Shanghai−test
HK−t	Hukou−test
YZ−t	Cultivated population−test
LDA	linear discriminant analysis
AMOVA	Analysis of molecular variance
